# Optimal Regimens and Cutoff Evaluation of Tildipirosin Against *Pasteurella multocida*

**DOI:** 10.3389/fphar.2018.00765

**Published:** 2018-07-26

**Authors:** Zhixin Lei, Qianying Liu, Yi Qi, Bing Yang, Haseeb Khaliq, Jincheng Xiong, Gopi Krishna Moku, Saeed Ahmed, Kun Li, Hui Zhang, Wenqiu Zhang, Jiyue Cao, Qigai He

**Affiliations:** ^1^State Key Laboratory of Agricultural Microbiology, College of Veterinary Medicine, Huazhong Agricultural University, Wuhan, China; ^2^Department of Veterinary Pharmacology, College of Veterinary Medicine, Huazhong Agricultural University, Wuhan, China; ^3^National Reference Laboratory of Veterinary Drug Residues and MAO Key Laboratory for Detection of Veterinary Drug Residues, Huazhong Agricultural University, Wuhan, China; ^4^Department of Pharmaceutics, College of Pharmacy, University of Minnesota, Minneapolis, MN, United States

**Keywords:** *Pasteurella multocida*, tildipirosin, pharmacokinetic/pharmacodynamic, epidemiological cutoff, PK-PD cutoff, optimal regimens

## Abstract

*Pasteurella multocida* (PM) can invade the upper respiratory tract of the body and cause death and high morbidity. Tildipirosin, a new 16-membered-ring macrolide antimicrobial, has been recommended for the treatment of respiratory diseases. The objective of this research was to improve the dose regimes of tildipirosin to PM for reducing the macrolides resistance development with the pharmacokinetic/pharmacodynamic (PK/PD) modeling approach and to establish an alternate cutoff for tildipirosin against PM. A single dose (4 mg/kg body weight) of tildipirosin was administered via intramuscular (i.m.) and intravenous (i.v.) injection to the pigs. The minimum inhibitory concentration (MIC) values of clinical isolates (112) were measured in the range of 0.0625–32 μg/ml, and the MIC_50_ and MIC_90_ values were 0.5 and 2 μg/ml, respectively. The MIC of the selected PM04 was 2 and 0.5 μg/ml in the tryptic soy broth (TSB) and serum, respectively. The main pharmacokinetic (PK) parameters including the area under the curve at 24 h (AUC_24 h_), AUC, terminal half-life (*T*_1/2_), the time to peak concentration (*T*_max_), peak concentration (*C*_max_), relative total systemic clearance (CL_b_), and the last mean residence time (MRT_last_) were calculated to be 7.10, 7.94 μg^∗^h/ml, 24.02, NA h, NA μg/ml, 0.46 L/h^∗^kg, 8.06 h and 3.94, 6.79 μg^∗^h/ml, 44.04, 0.25 h, 0.98 μg/ml, 0.43 L/h^∗^kg, 22.85 h after i.v. and i.m. induction, respectively. Moreover, the bioavailability of i.m. route was 85.5%, and the unbinding of tildipirosin to serum protein was 78%. The parameters AUC_24 h_/MIC in serum for bacteriostatic, bactericidal, and elimination activities were calculated as 18.91, 29.13, and 34.03 h based on the inhibitory sigmoid *E*_max_ modeling. According to the Monte Carlo simulation, the optimum doses for bacteriostatic, bactericidal, and elimination activities were 6.10, 9.41, and 10.96 mg/kg for 50% target and 7.86, 12.17, and 14.57 mg/kg for 90% target, respectively. The epidemiological cutoff value (ECV) was calculated to be 4 μg/ml which could cover 95% wild-type clinical isolates distribution. The PK-PD cutoff (CO_PD_) was analyzed to be 0.25 μg/ml *in vitro* for tildipirosin against PM based on the Monte Carlo simulation. Compared with these two cutoff values, the finial susceptible breakpoint was defined as 4 μg/ml. The data presented now provides the optimal regimens (12.17 mg/kg) and susceptible breakpoint (4 μg/ml) for clinical use, but these predicted data should be validated in the clinical practice.

## Introduction

*Pasteurella multocida* (PM) is a widespread pathogenic bacterium that can cause mucosal surfaces and respiratory tract infection in animals, which results in large economic losses in the livestock and poultry industry (Holst et al., 1992; Elazab et al., 2018). Furthermore, there are a few reports on the resistance of PM response to macrolide including tildipirosin, tilmicosin, tylosin, etc. (Andersen et al., 2012; Michael et al., 2012; Poehlsgaard et al., 2012). Therefore, it is essential for veterinarians to use antibiotics wisely in veterinary clinics.

Tildipirosin, a new 16-membered-ring macrolide, is a semisynthetic tylosin developed to treat respiratory pathogens, such as HPS, *P. multocida* (PM), APP, MH (Rose et al., 2013; Torres et al., 2016). With widespread use of macrolides, such as tylosin, tilmicosin, and tildipirosin in Europe, a new macrolide tildipirosin-resistant PM has emerged (Andersen et al., 2012; Olsen et al., 2015). Furthermore, tildipirosin was imported into China, and resistant strains were found in the clinical setting, breeding, and animal husbandry.

It is better to set the susceptibility of target bacteria to the antibiotics to reduce the development of bacterial resistance and strengthen the management of antibiotic use. According to the guidance of the VetCAST (an EUCAST sub-committee for veterinary antimicrobial susceptibility testing), the susceptibility cutoff values consisted of an ECV – the highest MIC (>95%) in the wild-type distribution; a PK/PD cutoff value (CO_PD_) – the most probable critical value (>90%) in the target population from the calculated PK/PD index such as AUC/MIC or terminal life (T) > MIC; and a clinical cutoff – the curving ratios for the antibiotic against the target bacterium if it is possible to implement and obtain the statistical data (Toutain et al., 2017). Based on the guidance of CLSI and EUCAST, there has not been a recommended susceptibility breakpoint for tildipirosin against PM. Therefore, it would be of great significance to establish such a criterion for both susceptibility testing and monitoring the development of resistance (Zhixin et al., 2017; Lei et al., 2018a).

Misuse and unreasonable dosage of antimicrobial agents were the main factors for the development of resistance (Burgess, 1999; Burgess et al., 1999; Wang et al., 2016). However, PK/PD integration modeling data can provide an optimal drug dosage strategy, reducing resistance development, which is a key method to evaluate the clinically relevant relationship between time, drug concentration, and effect. In the present study, tildipirosin recommended dosage (4 mg/kg body weight) has been anticipated by the European medicines agency (EMA, 2010) and has also been verified in different doses (2, 4, and 6 mg/kg body weight) according to the PK data (area under concentration-time curve from time, AUC_last_) in the previous report (Menge et al., 2012; Rose et al., 2013). However, there was no data that supported the recommended dosage (4 mg/kg body weight) under PD of tildipirosin against PM in China. This study investigated the PK and *in vitro* PD activity of tildipirosin in plasma obtained from healthy pigs, reviewed the recommended dosage, and formulated an optimum dose.

The objectives of the current study were (i) to assay the PD parameters and actions of tildipirosin against PM, (ii) to evaluate the PK properties of tildipirosin in plasma after i.m. administration, (iii) to formulate a rational dosage strategy and review the preceding recommended dosage based on PK/PD modeling for tildipirosin against PM, providing peak efficacy and marginal opportunity for the resistant development of PM (Jian et al., 2015; Xiao et al., 2015; Wang et al., 2016), and (iv) to establish the ECV and CO_PD_ of tildipirosin against PM based on the wild-type MIC distributions and PK/PD profiles data *in vitro* and *in vivo*.

## Materials and Methods

### Bacterial Strain Isolation

112 PM isolates were collected from pigs across China (mainly, Anhui, Hubei, Henan, Guangdong, and Jiangxi provinces) from 2013 to 2016. According to the MIC_90_ values of strains, a PM named PM04 strain whose minimal inhibitory concentration (MIC) was similar to MIC_90,_ was considered to find the antimicrobial activity of tildipirosin *in vitro*. *Escherichia coli* ATCC 25922 isolate was selected as the reference strain for determining antibiotic susceptibility. The isolate species were recognized by the method of PCR. Before testing MIC values, subculturing was performed for each sample in TSB and tryptic soy agar (TSA; Qingdao Hai Bo Biological Technology Co., Ltd., Shandong, China) containing 5% NCS (Zhejiang Tianhang Biotechnology Co., Ltd., Zhejiang, China) at least thrice. Tildipirosin was provided by Hubei Huisheng Biological Technology Company (Hubei, China).

### Determination of MIC and MBC

The MICs of 112 PM were measured with the process of agar dilution technique in line with the CLSI guidelines (CLSI, 2012; Zhang et al., 2016). Strains of PM (2–4 μl, about 10^8^ CFU/ml) were administrated onto TSA agar plates containing NCS, with twofold serial-dilutions of tildipirosin (0.0625–32 μg/ml). The plates with strains were nurtured in the atmosphere containing 5% CO_2_ for 48 h at 37°C. The MIC contained a minimum amount of tildipirosin where the visible growth of bacteria was inhibited.

The 100 μl suspension from the 96 well plates of PM04, of which MIC amount was estimated in broth dilution technique according to the CLSI guidelines, was subjected to 10-fold or more dilution with TSB, and then 10 μl of each diluted suspension was spread on the TSA plates and the colony counts were calculated after containing 5% CO_2_ for 48 h at 37°C. MBC was the minimum concentration of tildipirosin inhibiting 99.9% bacterial density of PM.

### ECV Determination for Tildipirosin Against PM

The resistance bacteria were excluded, and the wild-type PM should be defined for microorganisms which did not have any acquired resistance mechanisms to the tildipirosin. Additionally, the ECV calculated as the peak MIC for wild-type PM comprised over 95% in the MIC distributions according to the guidelines of CLSI and the previously described reports (Turnidge and Paterson, 2007; Pfaller et al., 2010, 2011; Lei et al., 2018a). The wild-type MIC distributions were checked and amended on the basis of standard distribution at the lower end of the MIC range for attaining the suitable distribution of MICs, which was executed with Sigma-Stat software (version 3.5, Systat Software Inc., United States). The mean and standard deviation of the normal distribution for the optimum non-linear least squares regression fitting MICs were assessed based on the software of GraphPad Prism (Version 7, GraphPad Software Inc., United States). The final ECV was calculated as the MIC value that captured over 95% of the optimum MIC distributions using the NORMINV and NORDIST functions in Microsoft Excel software was based on the preceding mean and standard deviation values. In other words, the ECV could be obtained using a summarized Excel form based on the above method.

### Time-Killing Curves *in Vitro* and *ex Vivo*

According to the MIC of tildipirosin against PM04 in MIC value, TSA plates were made with various concentrations of tildipirosin from the range of 1/4 to 32 MIC detailed in the previous study by Zhang and Lei and their companions (Zhang et al., 2016; Lei et al., 2018a). From the bacterial fluid, 100 μl was diluted with normal sterile saline (10^-1^ to 10^-5^ dilution ratio), then aliquots of the last four diluted samples were plunged onto the TSA plates at 0, 2, 4, 6, 8, 10, 12, and 24 h of culture, which were incubated in the atmosphere having CO_2_ for 48 h at 37°C.

Serum obtained from the pigs was considered as a culturemedium for the *ex vivo* growth of isolates, MIC, and time-killing curve. The determination methods were similar to *in vitro* protocol mentioned above utilizing the serum as a substitute for the TSB method. The bacteria (10^6^ CFU/ml) were co-incubated with the content of ileum samples collected from the pigs at various time intervals (0, 0.25, 1, 2, 4, 12, and 24 h) after supplementation with 4 mg/kg tildipirosin by i.m. route of administration. The *ex vivo* time-killing curve was fitted to a PD model with the theory of a decrease in tildipirosin concentration based on incubation time with inhibitory sigmoid *E*_max_ model.

### PK Study

#### Animals

Eight healthy pigs (of both sexes), weighing 15–20 kg and 4–5 weeks, were selected for this research work. These healthy animals were kept in a separate pen with free and adequate water availability, and no antibiotic feed was permitted. Moreover, animals were offered feed and water for 7 days to acclimate prior to the testing. After 7 days, pigs were intravenously (i.v.) injected with a single dose of 4 mg/kg tildipirosin. After a period of radical washing for approximately 2 weeks, the pigs were injected i.m. with the same dose of tildipirosin.

All animal experiments were permitted by Laboratory Animal Use and Care Committee in Hubei Science and Technology Agency (permit number SYXK 2013-0044) and executed according to the guidelines of committee. The anesthetics were given to reduce the pain and adverse effects in animals.

### Collection of Plasma Samples and HPLC Analysis

Three ml of blood samples were collected at 15 and 30 min, and then at various time points – 1, 2, 4, 6, 8, 10, 12, 24, 48, and 72 h, after i.v. and i.m. administrations. The obtained plasma samples were immediately freezed and then centrifuged at about 3000 *g* for 10 min before preserving them at -80°C until further investigation.

A C18 reverse-phase column (250 mm × 4.6 mm, i.d., 5 μm, Agilent, United States) was used for HPLC, which was performed with a 289 nm detection wavelength at 30°C. Plasma (0.5 ml) samples were mixed with 200 μl dipotassium hydrogen phosphate solution (0.1 mol/L), and then were extracted twice with 5 ml diethyl ether. The supernatants were attained by the centrifugation method, and then vaporized to dryness under the nitrogen chamber at the water bath kettle with 45°C. The final samples were re-suspended with the mobile phase by the initial volume (0.5 ml). The samples for tildipirosin concentration determination were analyzed using HPLC method, which had been optimized by our laboratory in the previously described report (Lei et al., 2018a) within a month. The PK data were analyzed with Phoenix WinNonlin 6.1 software (Pharsight Co., Ltd.).

### Binding of Tildipirosin to Serum Protein

The serum protein binding of tildipirosin was determined on the collected samples at different points from the eight pigs used in this study. The total concentration of tildipirosin was detected in every sample. The samples were redetermined after filtration through dialysis tubing (Thermo Fisher Scientific Co., LTD., United States).

### PK/PD Integration Analysis

Although most macrolides were classified as time-dependent killing drugs, tildipirosin was concentration-dependent, and the PK/PD index were the AUC_24 h_/MIC and the *C*_max_/MIC (Mouton et al., 2002; Chigutsa et al., 2012; Rose et al., 2013; Xiao et al., 2015). The AUC_24 h_/MIC and *C*_max_/MIC were considered as paired PK/PD parameters which were calculated in each dose of the time-killing curve. The inhibitory sigmoid *E*_max_ model was applied to evaluate the assimilation of correlation of AUC_24 h_/MIC ratio *in vitro* and bacteria count change (CFU/ml) in serum during 24 h incubation with WinNonlin software (Aliabadi and Lees, 2000, 2001, 2002; Sidhu et al., 2011). The model equation was described as follows in equation 1.

(1) $$\begin{array}{c} E=E_{\max\;}\cdot (1-\frac{C^{N}}{C^{N}{+EC}_{50}^{N}}) \end{array}$$

*E*, presented the effect of antimicrobial agent counted as log_10_ difference of bacterial number before and after the 24 h incubation *in vitro*; *E*_max_, measured the deviations in log_10_ difference between 0 and 24 h in the control and tildipirosin samples; EC_50_, the AUC_24_/MIC value reached 50% of the *E*_max_; C, presented the AUC_24_/MIC ratio; *N*, presented the Hill coefficient.

### Dose Estimations

The given formula was performed to calculate the doses in different magnitudes of efficiency containing (*E* = 0, no change in bacterial count, *E* = -1, 99.9% reduction in the count, *E* = -3, 99.99% reduction) for estimating an optimum regimen.

(2) $$\begin{array}{c} Dose=\frac{(AUC/MIC)\cdot MIC_{90}\cdot CL}{fu\cdot F} \end{array}$$

*AUC/MIC*, meant the targeted endpoint for optimal efficacy; *MIC*, meant minimum inhibitory concentration; *CL*, meant clearance per day; *fu*, meant the free fraction of drug in plasma, ignoring if there was minimal binding; *F*, meant the bioavailability.

The distribution probabilities for predicted daily dosage were performed to achieve simulated 50 and 90% TAR under 10,000 trails with Crystal Ball software (version 7.2.2, Oracle, United States) for bacteriostatic, bactericidal, and elimination activities.

### Monte Carlo Analysis and PK/PD Cutoff Calculation

The Monte Carlo simulation (including 10,000 iterations) was performed using the Crystal Ball software (version 7.2.2, Oracle, United States) based on the selected PK/PD target index (AUC_24_/MIC, *E* = -3, bactericidal activity) (Lei et al., 2017a,b,c, 2018b). The CO_PD_ was calculated as the MIC when PTA reached up to 90% based on the CLSI guidelines and other previously described studies (Turnidge and Paterson, 2007; Lei et al., 2018a).

### Statistical Analysis

MIC_90_ was measured by using statistical package of SPSS software, and statistical analysis was executed with Student’s *t*-test. Significant differences were checked using *p* < 0.05.

## Results

### MIC and MBC Determination Both *in Vitro* and *ex Vivo*

**FIGURE 1:**
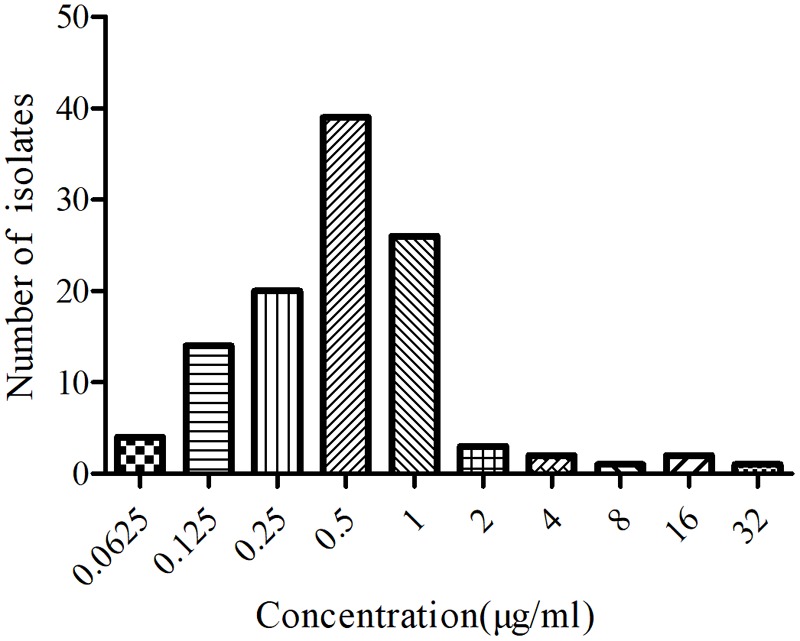
The MIC distributions of tildipirosin against PM (112 clinical isolates).

The MIC distributions of tildipirosin against 112 clinical isolates of PM were displayed in **Figure 1**, of which MIC values ranged from 0.0625 to 32 μg/ml. MIC_50_ and MIC_90_ of the distribution were 0.5 and 2 μg/ml, respectively, calculated with SPSS Statistics version 17.0, evincing that tildipirosin had a potent antibacterial efect on PM (**Table 1**).

**Table 1:** Minimum inhibitory concentration (MIC) and MBC (μg/ml) of tildipirosin against PM04 *in vitro* and *ex vivo*.

Target strain	MIC	MBC	MIC_50_	MIC_90_
TSB	2	8	0.5	2
Serum	0.5	1	–	–

According to the MIC_*90*_ value, a clinical isolate and PM04 whose MIC was similar to MIC_*90*_ was selected to explore the antimicrobial activity of tildipirosin *in vitro*. The MIC of tildipirosin against PM04 was 0.5 μg/ml which was 0.25 times lower than that (2 μg/ml) in TSB. The MBC of tildipirosin against PM04 in TSB and serum were 8 and 1 μg/ml, respectively (**Table 1**). The ratios of MBC/MIC *in vitro* (TSB) and *ex vivo* (serum) were 2 and 4, respectively. Which indicated that tildipirosin might have a strong bacteriostatic activity both *in vitro* and *ex vivo*, and concentration-dependent profile against PM.

### ECV Calculation

**FIGURE 2:**
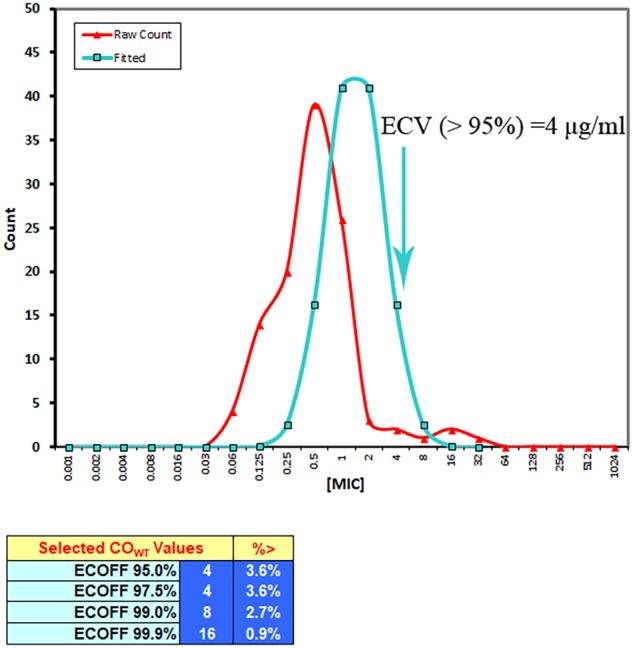
The ECV evaluation using the statistical program “ECOFFinder.”

The MIC distributions of tildipirosin against clinical PM were presented in **Figure 1**. According to the guidance of CLSI and the previously described reports (Turnidge and Paterson, 2007; Pfaller et al., 2011; Lei et al., 2018a), the resistant bacteria among the wild-type clinical isolates should be removed, therefore the three isolates whose MICs were over 16 μg/ml and detected to be the resistant bacteria should be discarded as reported by Michael et al. (2012). The wild-type MIC distributions were statistically detected by the calculation of over 95% ECV plotted in **Figure 2** using the freeware statistical program “ECOFFinder” which could estimate the wild-type population and derive the ECV value^1^. This software could simplify the operations and calculation procedures easily. The evaluated MICs whose values were 4, 8, and 16 μg/ml could encompass 97.5, 99, and 99.9% of the wild-type isolates. Finally, the probability of the MIC at 4 μg/ml encompassed over 95% of the wild-type isolates and was defined as the ECV (**Figure 2**).

### Antimicrobial Activity *in Vitro* and *ex Vivo*

**FIGURE 3:**
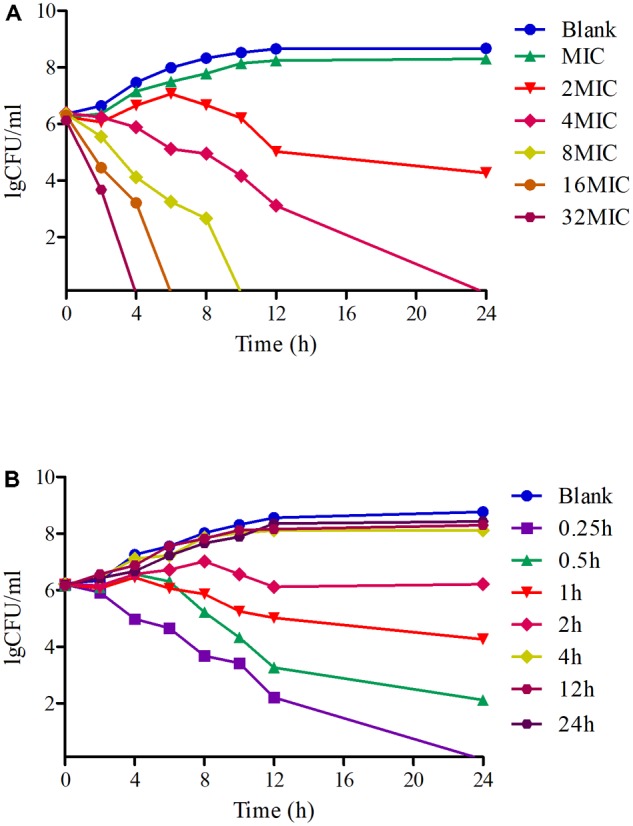
Time-killing curves of tildipirosin against PM04 in the TSB and serum. **(A)** Means the curve in the TSB (*vitro*), **(B)** means the curve in the serum (*ex vivo*).

Time-killing curves of tildipirosin against PM04 *in vitro* were illustrated in **Figure 3**. According to the curves profiles, tildipirosin displayed a concentration-dependent characteristic, a more rapid bactericidal activity with an increase of drug concentration. It acted intensively with bacteriostatic effect when the tildipirosin concentrations were higher than 2 MIC *in vitro* (TSB), while the concentration at 0.25 h (1 μg/ml, 2 MIC) could eradicate PM04 completely *in vivo* (serum) (**Figure 3**). These results were similar to the MBC determination, but the difference of antimicrobial activity in TSB and serum might attribute to the effect of serum, which could have a positive antibacterial effect.

### PK of Tildipirosin

**FIGURE 4:**
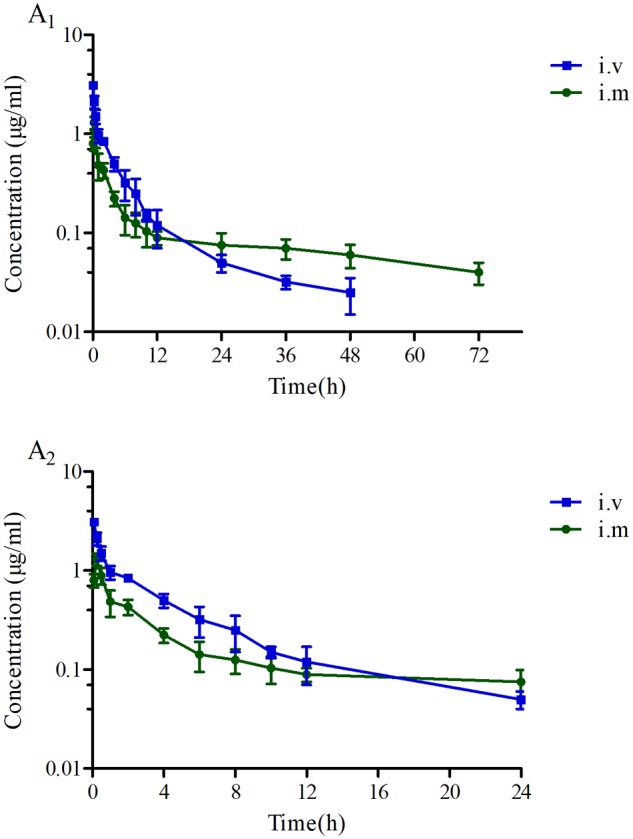
The concentration-time curves of tildipirosin at a dose of 4 mg/kg after i.v. and i.m. administrations. **(A_1_)** Presented the concentration-time during the whole process, **(A_2_)** presented the concentration-time within 24 h.

The proposed HPLC method for tildipirosin was according to the previously described research in this lab and the detailed introduction is referred to in the report by Lei et al. (2018a). The PK parameters of tildipirosin in the serum after i.v. and i.m. administrations at a dose of 4 mg/kg were presented in **Table 2**. The PK parameters were derived by non-compartmental analysis. The PK parameters of tildipirosin in the serum were measured using WinNonlin software. The results for the AUC_24 h_, AUC, *T*_max_, *T*_1/2_ of tildipirosin, *C*_max_, CL_b_, and the mean resistance time (MRT) in the serum after i.v. and i.m. administrations were shown in **Table 2**. The bioavailability for tildipirosin after i.m. administration was determined as 85.5% (**Table 2**). The mean ± SD of tildipirosin concentration-time profiles were shown in **Figure 4** after i.v. and i.m. administrations, respectively. Additionally, the percentage of free tildipirosin concentration with unbinding protein in the plasma was 78.00 ± 5.23% from the eight pigs used in this study.

**Table 2:** Pharmacokinetic parameters in plasma after 4 mg/kg i.v. and i.m. administration, respectively.

Parameters	Units	i.v	i.m
AUC_24 h_	μg^∗^h/ml	7.10 ± 0.91	3.94 ± 0.62
AUC	μg^∗^h/ml	7.94 ± 1.11	6.79 ± 0.45
*T*_max_	h	–	0.25 ± 0.06
*T*_1/2_	h	24.02 ± 3.12	44.04 ± 4.56
*C*_max_	μg/ml	–	0.98 ± 0.11
CL_b_	L/h	0.46 ± 0.08	0.43 ± 0.07
MRT_last_	h	8.06 ± 1.6	22.85 ± 2.96
*F*	%		85.5 ± 7.11

*AUC_*24 h*_, the area under the curve within 24 h; AUC, the area under the curve; T_*max*_, the time to peak concentration; T_*1/2*_, the eliminate half-life; C_*max*_, the peak concentration; CL_*b*_, relative total systemic clearance; MRT, the mean residence time; F, the bioavailability.*

### PK/PD Integration and Modeling

**FIGURE 5:**
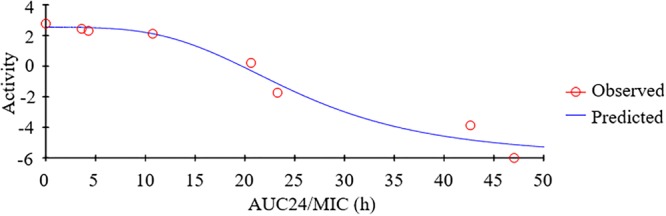
Plots of *ex viv*o AUC_24 h_/MIC ratios versus the amount of difference within 24 h.

As a concentration-dependent antibiotic, the selected PK/PD parameters attained from PK data *in vivo* combined with MIC *ex vivo* were presented in the **Table 3**. The ratios of *C*_max_/MIC, AUC_24h_/MIC and T > MIC were 1.96, 7.88, and 0.97 h, respectively, on the basis of PK/PD data *ex vivo* (**Table 2**). *Ex vivo* antibacterial activity of tildipirosin against PM04 was measured in serum samples collected before and at 0.25, 0.5, 1, 2, 4, 12, and 24 h after i.m. induction. The relationship between antimicrobial efficiency and the *ex vivo* PK/PD parameter of AUC_24h_/MIC ratios were simulated by using the inhibitory sigmoid *E*_max_ model. The model parameters of the Hill coefficient N, *E*_max_, and AUC_24h_/MIC values are presented for three levels of growth inhibition in **Table 4** and **Figure 5**. The values of the AUC_24h_/MIC ratio required for bacteriostatic activity (*E* = 0), bactericidal activity (*E* = -3), and bacterial elimination (*E* = -4) were 18.91, 29.13, and 34.03 h, as presented in **Table 4**.

**Table 3:** The parameters of PK/PD integration of tildipirosin.

Parameters	Units	Mean ±*SD*
AUC_24h_/MIC	h	7.88 ± 0.87
*C*_max_/MIC	–	1.96 ± 0.21
*T* > MIC	h	0.97 ± 0.10

**Table 4:** The main *ex vivo* parameters of PK/PD modeling of tildipirosin in plasma.

Parameters	Units	Mean ±*SD*
*E*_max_	LgCFU/ml	2.54 ± 0.37
EC_50_	h	23.52 ± 1.80
*N*	–	3.48 ± 0.81
AUC_24 h_/MIC for bacteriostatic (*E* = 0)	h	18.91 ± 1.32
AUC_24 h_/MIC for bactericidal (*E* = -3)	h	29.13 ± 2.44
AUC_24 h_/MIC for eradication (*E* = -4)	h	34.03 ± 4.11

*E_*max*_, presented the Lg change in bacterial counts of blank sample; EC_*50*_, presented the value to achieve 50% maximal antibacterial effect; N, presented the Hill coefficient.*

### PK/PD Cutoff Calculation of Tildipirosin Against PM04

The calculated cumulative target achievement for PK/PD parameter (AUC_24 h_/MIC) in serum (*ex vivo*) was 29.13 ± 2.44 h, with assured bactericidal activity (*E* = -3). The PTA values were calculated as 0, 0, 68.64, and 100% when the MIC values were defined as 0.5, 0.25, 0.125, and 0.0625 μg/ml, respectively, after a single 4 mg/kg i.m. was administrated in pigs (**Table 5**). As a result, a PTA ≥ 90% could be achieved for isolates with MIC ≤ 0.0625 μg/ml in the serum after i.m. injection at a dose of 4 mg/kg body weight (**Table 5**). Furthermore, it could be deduced to be 0.25 μg/ml in TSB (*in vitro*). Therefore, the CO_PD_ of tildipirosin against PM could be defined as 0.25 μg/ml *in vitro*.

**Table 5:** The AUC_24_/MIC values calculated with Monte Carlo simulation for PTA.

Doses	Effect	MIC (μg/ml)			
		**0.0625^∗^**	**0.125**	**0.25**	**0.5**

4 mg	Eradication	100%	68.64%	0%	0%

*^∗^Represents the value of PK/PD cutoff breakpoint.*

### Estimation of Dosages

**FIGURE 6:**
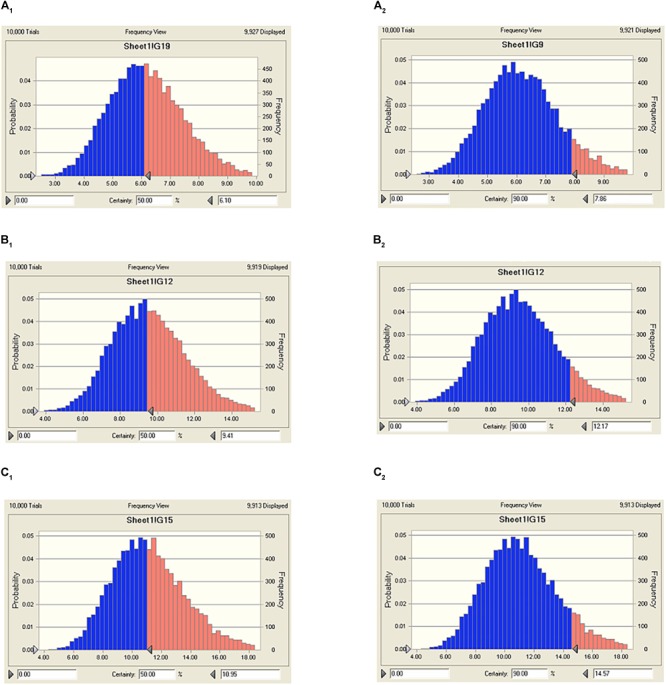
The predicted regimes of tildipirosin curing PM for 50% and 90% TAR. **(A_1_–C_1_)** Meant the predicted population doses for bacteriostatic, bactericidal, and elimination activities for 50% TAR; **(A_2_–C_2_)** meant the predicted population doses for bacteriostatic, bactericidal, and elimination activities for 90% TAR.

The predicted daily doses were given in **Table 6** based on AUC_24 h_/MIC ratios and CL_b_ for these three levels of antibacterial activity measured from the PK/PD integrating model and the distribution of *ex vivo* MIC using Monte Carlo Simulations in Oracle Crystal Ball. The distributions of predicted population dose (AUC_24 h_/MIC) values of tildipirosin curing PM for 50 and 90% targets were observed, respectively, and illustrated in **Figure 6**. In this research, based on the dose equations, the predicted doses for bacteriostatic, bactericidal, and elimination activity of tildipirosin against PM over 24 h were 6.10, 9.41, and 10.96 mg/kg.bw for 50% target, respectively, and 7.86, 12.17, and 14.57 mg/kg.bw for 90% target, respectively, in **Table 6**.

**Table 6:** The predicted daily doses of tildipirosin curing PM.

Predicted doses (mg/kg.bw)	Target ratios	
	50%	90%
Bacteriostatic (*E* = 0)	6.10	7.86
Bactericidal (*E* = -3)	9.41	12.17
Eradication (*E* = -4)	10.96	14.57

## Discussion

As the newest macrolide antibiotic, tildipirosin has a long-acting, strong bacteriostatic action, high bioavailability and drug concentrations particularly in lung tissue and other outstanding profiles (Rose et al., 2013; Lei et al., 2018a). In the current study, the MICs of 112 clinical isolates (PM) from across the country of China were monitored according to the CLSI M07-A9 guidance document. The MIC concentrations of tildipirosin against PM were in the range of 0.0625–32 μg/ml containing three resistant bacteria with MICs over or equal to 16 μg/ml, in possession of resistance genes erm (42) or msr (E) and mph (E) in this study. These resistant genes for PM to tildipirosin have been demonstrated in the previously described reports by Jacob, Lei, and Geovana, respectively (Michael et al., 2012; Poehlsgaard et al., 2012; Lei et al., 2018a). The resistance of PM to macrolides including tildipirosin was considered as more and more serious. Furthermore, the control of PM was difficult due to its high resistance and rapid spread to common antimicrobial drugs resulting from the overuse and misuse (Tang et al., 2009; Ferreira et al., 2012). Therefore, it is necessary to establish an optimal scheme to effectively reduce the PM resistance development.

In the current study, the MIC_50_ and MIC_90_ of the 112 clinical isolated PM across China were tested to be 0.5 and 2 μg/ml *in vitro*, respectively. The isolated PM04 with the high toxicity gene (toxA) from Anhui province was selected as the respective PM for the further study, and the MIC of the PM04 was 2 μg/ml in TSB, similar to the value of MIC_90_ (**Table 1**). Additionally, the MIC of the PM04 *in vivo* (serum) was detected to be 0.5 μg/ml (**Table 1**), which is 0.25 times lower than that *in vitro*. The difference (MICs) between TSB and serum might be mainly due to the serum effect which was also reported in the previous studies by Toutain et al. (2016) and Lei et al. (2017b). In most of the previously published reports, the clinical isolates were randomly selected for PK/PD analysis (Robertson et al., 2005; Haritova et al., 2006; Nedelman et al., 2007; Sidhu et al., 2010). However, in this study, the PM04 was the most representative clinical isolate with high toxicity (which has been demonstrated in Kunming mice, but not presented in this study) and high MIC value similar to the MIC_90_ of the population (PM). Therefore, it could be more reliable for further study.

Based on the guidelines of CLSI, it is usually encouraged that the susceptibility of wild-type bacteria to antibiotics should be tested prior to their use for treatment (Lei et al., 2018a). There has not been any susceptibility breakpoint standard for tildipirosin against PM. The ECV of tildipirosin against PM was calculated to be 4 μg/ml in this study, which was the first declared result and the ECV was calculated using the integrated software “ECOFFinder” which could simplify the procedures and progresses. The ECOFFinder was also applied in the report published by Lees et al. (2015). Compared with the previously published study by Lei et al. (2018a), the ECV of tildipirosin against HPS was 8 μg/ml which is two times higher than that (4 μg/ml, PM) in this study. The difference of these two bacteria might attribute to the wild-type MIC ranges: the MIC of tildipirosin against HPS was in the range of 0.03125–256 μg/ml, while the MIC range of tildipirosin against PM was from 0.06125 to 32 μg/ml. Moreover, the species difference might also be another reason. The CO_PD_ of tildipirosin against PM was calculated to be 0.25 μg/ml in the TSB (**Table 5**). Although the susceptibility breakpoint was defined based on the three cutoff values including ECV, CO_PD_ and clinical cutoff, the last cutoff was required to monitor in the clinic (Toutain et al., 2017). According to the guidelines of CLSI and EUCAST, the final breakpoint could be recognized as 4 μg/ml without the data from clinical investigative findings. The result of susceptibility breakpoint (4 μg/ml) provided from this study could be regarded as an alternative for isolated strains MIC determination in clinical veterinary medicine.

The mean concentrations of tildipirosin over the MIC (0.5 μg/ml) for PM in the serum (*T* > MIC) sustained 0.97 h (**Table 3**), demonstrating that tildipirosin had a strong and long-lasting bacteriostatic activity against PM which was similar to HPS (4 h, *T* > MIC) in the previous reports (Rose et al., 2013; Lei et al., 2018a). Additionally, the means of the AUC_24 h_ (3.94 μg^∗^h/ml), *C*_max_ (0.98 μg/ml), and CL_b_ (0.43 L/h) in this study were similar to those (4.25 μg^∗^h/ml, 1.01 μg/ml, and 0.28 L/h) in the published report by Lei et al. (2018a). The PK profiles for tildipirosin in pigs after i.v. administration were investigated for the first time, and the absolute bioavailability of i.m. administrated tildipirosin was calculated to be 85.5% which was similar to the study on the cattle (78.9%) reported by Menge et al. (2012) and Rose et al. (2013). The serum protein binding ratio was tested to be 22% which was similar to the report of EMA (30%) (EMA, 2010). These results demonstrated that tildipirosin had a high extent of absorption and low-level of protein binding effect and could be proposed widely for use in the veterinary clinic practice.

Based on the killing-time curve profiles of tildipirosin against PM04 (**Figure 3**) *in vitro* and *ex vivo*, it was obvious that tildipirosin presented the concentration-dependent action and the parameter “AUC_24 h_/MIC” was generally regarded as the threshold for the successful therapeutic outcome of macrolides (Lei et al., 2017b, 2018b,c). In the previously published studies (Sang et al., 2016; Wang et al., 2016; Zhang et al., 2016; Lei et al., 2017a, 2018a), the AUC_24 h_/MIC > 30 h, and AUC_24 h_/MIC > 125 h were used frequently for macrolides and fluoroquinolones against Gram-negative bacteria; these thresholds might be different for different drugs, against different kinds of bacteria due to the differences in the immune status of target animals and pathogens (Toutain et al., 2002; Ahmad et al., 2015). Therefore, it is essential to obtain the exclusive PK/PD target for tildipirosin against PM (Lei et al., 2018c). In this study, the PK/PD target (AUC_24 h_/MIC) for tildipirosin against PM was 29.13 h when it acted on the bactericidal activity (*E* = -3) using PK/PD integration modeling (**Table 4**). This target value was much higher than the original parameter (7.88 h) (AUC_24 h_/MIC) in **Table 3**; this result revealed that the current dosage (4 mg/kg) might not reach the bactericidal effect of tildipirosin against PM. Therefore, the optimal dosage regimens are extremely necessary. Based on the *ex vivo* PK/PD modeling using inhibitory sigmoidal *E*_max_ model, a favorable correlation (*R*^2^= 0.988) was shown between the PK/PD index (AUC_24 h_/MIC) and predicted antibacterial efficacy which could be observed in **Figure 5**. Furthermore, the predicted population daily dosages over 24 h for tildipirosin against PM acting bacteriostatic, bactericidal, and eradication activities were calculated as 6.10, 9.41, and 10.96 mg/kg for 50% TAR, and 7.86, 12.17 and 14.57 mg/kg TAR, respectively (**Figure 6** and **Table 6**) according to the Monte Carlo simulation which is an effective method to adjust the dosage regimen for clinical use (Nielsen and Friberg, 2013; Dorey et al., 2017). Therefore, it was suggested that 12.17 mg/kg could guarantee the clinical efficacy and bactericidal action in this study. Due to the bacterial endpoint, *in vivo* might differ from the predicted dosages and the target animals’ immune system could also affect the bacterial eradication action of drug, and the collected animals’ samples (a small scale) in this study were not enough to strongly support the conclusions. The estimated population daily dosages should be validated in future for veterinary clinical practice and research.

## Conclusion

The intelligent use of antibiotics was increasingly important in the veterinary clinic, and the misuse and abuse of antibiotics were the uppermost reason for the bacteria resistance development (Nguyen et al., 2012; Lei et al., 2018c). The current study firstly provided the susceptibility breakpoint (4 μg/ml) which could distinguish the resistance bacteria easily and clearly and be regarded as a kind of resistance standard for tildipirosin against PM in further studies. Furthermore, the finding of this study also proved that the current dosage 4 mg/kg tildipirosin (AUC_24 h_/MIC = 7.88 h) could not cover and guarantee the bactericidal effect, while the predicted daily dosage over 24 h (12.17 mg/kg) might be sufficient and effective (**Table 6**). In conclusion, the susceptibility breakpoint and predicted daily dosage from this study have to be further validated in clinical practice.

## Author Contributions

JC and QH conceived this study. QL and ZL designed the experiments. ZL, YQ, KL, HZ, and BY performed the experiments. ZL wrote the manuscript. QH, HK, SA, JX, GM, WZ, and JC improved the language. All authors reviewed the manuscript.

## Conflict of Interest Statement

The authors declare that the research was conducted in the absence of any commercial or financial relationships that could be construed as a potential conflict of interest.

## Footnotes

1 http://www.uphs.upenn.edu/bugdrug/antibiotic_manual/ECV.htm

## Abbreviations

APP: *Actinobacillus pleuropneumoniae*
AUC: the area under the curve
AUC_24h_: area under the curve at 24 h
CFU: colony forming unit
CL_b_: relative total systemic clearance
CLSI: Clinical and Laboratory Standard Institute
*C*_max_: peak concentration
CO_PD_: PK/PD cutoff
ECVs: epidemiological cutoff values
EMA: European medicines agency
EUCAST: European Committee on Antimicrobial Susceptibility Testing
HPLC: high-performance liquid chromatography
HPS: *Haemophilus parasuis*
i.m.: intramuscular
i.v.: intravenous injection
MBC: minimal bactericidal concentration
MH: *Mannheimia haemolytica*
MIC: minimum inhibitory concentration
MRT: the mean residence time
NCS: newborn calf-serum
PCR: polymerase chain reaction
PD: pharmacodynamic
PK: pharmacokinetic
PK/PD: pharmacokinetics/pharmacodynamics
PM: *Pasteurella multocida*
PTA: the probability of target attainment
*T*_1/2_: terminal half-life
TAR: target achievement ratio
*T*_max_: the time to peak concentration
TSB: tryptic soy broth
VetCAST: Veterinary Committee on Antimicrobial Susceptibility Testing
